# Effects of S-1 combined with radiotherapy in the treatment of advanced esophageal cancer: A systematic review and meta-analysis protocol

**DOI:** 10.1097/MD.0000000000010164

**Published:** 2018-03-23

**Authors:** Wei Wang, Dong Xing, Yingjian Song, Feiyu Liu

**Affiliations:** a Department of Thoracic Surgery, The Affiliated Yantai Yuhuangding Hospital of Qingdao University, Yantai, Shandong, China; b Department of Thoracic and Cardiovascular Surgery, The University of Texas MD Anderson Cancer Center, Houston, TX, USA; c Department of Radiology; d Department of Pharmacy, The Affiliated Yantai Yuhuangding Hospital of Qingdao University, Yantai, Shandong, China.

**Keywords:** chemoradiotherapy, CRT, esophageal neoplasms, S-1

## Abstract

**Background:**

Esophageal cancer is one of the worst malignant digestive neoplasms with poor treatment outcomes. Definitive concurrent chemoradiotherapy (CRT) has become the standard nonsurgical treatment option for locally advanced esophageal cancer. The chemotherapeutic drugs 5-fluorouracil and cisplatin have been most commonly used in CRT of esophageal cancer. However, radiotherapy combined with 5-FU/cisplatin often delivers severe toxicity to patients. S-1 as an oral chemotherapeutic drug exhibits higher anti-tumor activity, less adverse effects, and better biological availability. S-1 also has excellent effects as a CRT regimen for esophageal cancer.

**Methods:**

A systematic literature search will be performed through January 2018 using MEDLINE, EMBASE, the Cochrane Central Register of Controlled Trials, and Google Scholar for relevant articles published in any language. Randomized controlled trials, prospective comparative studies will be included. All meta-analyses will be performed using Review Manager software. The quality of the studies will be evaluated using the guidelines listed in the Cochrane Handbook. The Preferred Reporting Items for Systematic Reviews and Meta-Analyses statements will be followed until the findings of the systematic review and meta-analysis are reported.

**Results:**

The results of this systematic review and meta-analysis will be published in a peer-reviewed journal.

**Conclusion:**

Our study will draw an objective conclusion of the effects of S-1 combined with radiotherapy in the treatment of unresectable esophageal cancer and provide level I evidence for clinical decision makings.

## 1 Introduction

Esophageal cancer is the ninth most commonly diagnosed cancer and the sixth most common cause of cancer-related deaths worldwide in 2013.^[1]^ Moreover, it is one of the worst malignant digestive neoplasms with poor treatment outcomes. Esophagectomy plays an important role and offers a potential curable chance for the early stage of esophageal cancer.^[2–4]^ But at initial diagnosis, about 40% to 60% of patients are not candidates for surgical treatment.^[5]^ Definitive concurrent chemoradiotherapy (CRT) has become the standard nonsurgical treatment option for locally advanced esophageal cancer, based on the evidence of early trials.^[6,7]^ And even some studies have shown comparable efficacy of definite CRT compared to surgery in patients with nonmetastatic disease.^[8,9]^ The chemotherapeutic drugs 5-fluorouracil (5-FU) and cisplatin have been most commonly used in the definitive CRT of esophageal cancer.^[10]^ However, radiotherapy combined with 5-FU/cisplatin chemotherapy often delivers severe toxicity to patients.^[11–13]^ A retrospective study that investigated the toxicity of CRT with 5-FU and cisplatin showed the treatment-related mortality rate was as high as 18% in elderly patients (age≥75 years).^[14]^ Therefore, exploring new CRT regimens with better tolerance and lower toxicity for patients with esophageal cancer are desperately needed.

S-1 is a novel oral combination drug comprising tegafur (FT), a prodrug of 5-FU, and 2 modulators of 5-FU metabolism, gimeracil (CDHP) and oteracil (Oxo), in a 1:0.4:1 molar ratio (FT:CDHP:Oxo). Evidence suggested that orally administered S-1 that mimics continuous infusion of 5-FU exhibits higher antitumor activity, less adverse effects, and better biological availability while compared with conventional 5-FU.^[15–17]^ In recent years, S-1 as an excellent CRT drug, single use or combined with platinum, has been widely applied for advanced esophageal cancer and achieved good clinical remission rate (CR).^[18–23]^ However, the sample size of these studies was relatively small and results in weak statistical power. Therefore, we conduct a systematic review and meta-analysis related to S-1-based CRT versus radiotherapy alone in the treatment of advanced esophageal cancer to further evaluate the clinical value of S-1. Moreover, in order to minimize the heterogeneity and bias, we will select randomized controlled trials (RCTs) and prospective comparative studies. The evidence grade will be determined by using the guidelines of the Grading of Recommendations, Assessment, Development, and Evaluation (GRADE) system. If data are sufficient, we will also conduct subgroup analyses using different histological types.

## 2 Objective

A systematic review and meta-analysis will be performed to assess the efficacy and safety of S-1 combined with radiotherapy in the treatment of unresectable esophageal cancer.

## 3 Methods

This protocol for systematic review and meta-analysis is performed according to the Preferred Reporting Items for Systematic Review and Meta-Analysis Protocols (PRISMA-P) statement.^[24]^ This protocol has been registered in the PROSPERO network (registration number: CRD42018088277). The systematic review and meta-analysis will be reported according to the Preferred Reporting Items for Systematic Reviews and Meta-Analyses (PRISMA) guidelines.^[25]^

### 3.1 Eligibility criteria

#### 3.1.1 Types of participants

The included participants will be adults who were diagnosed with advanced esophageal cancer histologically or cytologically confirmed and treated with radiotherapy. Comparisons of S-1-based CRT with radiotherapy alone in the clinical treatment were evaluated. There will be no restrictions regarding sex, race/ethnicity, education and economic status, and no restriction in publication language.

#### 3.1.2 Types of studies

We propose to include studies that report comparisons between S-1-based CRT and radiotherapy alone in the treatment of advanced unresectable esophageal cancer. RCTs and prospective comparative studies will be used for the qualitative and quantitative synthesis of the systematic review.

#### 3.1.3 Exclusion criteria

Non-peer reviewed articles, review articles, case reports, case series, animal studies, meeting abstracts, letters to the editor, commentaries, editorials, proceedings, and other nonrelevant studies will be excluded from analysis.

### 3.2 Information sources

We will perform a systematic literature search through January 31, 2018 using MEDLINE, EMBASE, the Cochrane Central Register of Controlled Trials, and Google Scholar for relevant articles published in any language.

### 3.3 Search strategy

The relevant searching terms will match Medical Subject Heading terms, and the searches will be repeated immediately before the final analyses to identify additional studies for inclusion. An example of the PubMed search strategy is shown in Table 1.

**Table 1:**
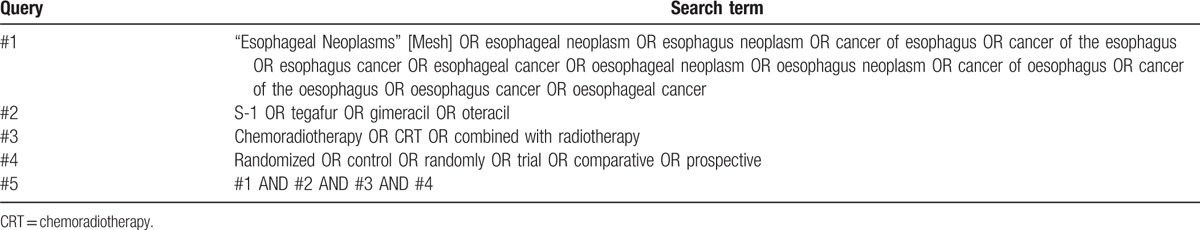
Search strategy for PubMed.

### 3.4 Study records

#### 3.4.1 Selection of studies

Two review authors (WW and DX) will independently screen titles and abstracts of all the potential studies to assess whether they meet the inclusion criteria as defined by the protocol. We will retrieve the full text of all potentially eligible studies and 2 review authors (WW and DX) will independently screen the full text and identify studies for inclusion, and record reasons for exclusion of the ineligible studies. Any disagreement will be resolved through discussion or, if required, consultation with a third review author (YS or FL). Duplicates will be excluded and multiple reports of the same study will be integrated into 1 unit of interest in the review. The selection process will be recorded in sufficient detail to complete a PRISMA flow diagram and “Characteristics of excluded studies” table.^[26]^ No language restrictions will be imposed.

#### 3.4.2 Data extraction and management

Data will be extracted from the included studies by 3 authors (WW, DX, and YS) independently and recorded on a predesigned data collection form. We will extract the following study characteristics:

(1) *Study characteristics*: study design, number of study centers and locations, study setting, withdrawals, total duration of the trial, periods of data collection, follow-up duration, and blanking periods.
(2) *Population characteristics*: inclusion and exclusion criteria, number, mean age, age range, gender, diagnostic criteria, pathological confirmation, staging of the tumor according to the AJCC TNM classification for esophageal cancer.
(3) *Intervention characteristics*: total radiation dose, fractions, S-1 dose, administration frequency, and cycles.
(4) *Outcomes*: primary and secondary outcomes specified and collected, and time points reported.

### 3.5 Outcomes

#### 3.5.1 Primary outcome

The primary outcome measure of our systematic review is overall survival (OS).

#### 3.5.2 Secondary outcomes

The secondary outcomes are: progression-free-survival (PFS), objective response rate (ORR), and grade 3 and 4 adverse events.

### 3.6 Assessment of risk of bias

Three review authors (WW, DX, and YS) will independently assess the risk of bias for each study using the criteria outlined in the Cochrane Handbook for Systematic Reviews of Interventions. Any disagreements will be resolved by discussion or by involving another review author (FL). The risk of bias will be assessed according to the following domains: random sequence generation; allocation concealment; blinding of participants and personnel; blinding of outcome assessment; incomplete outcome data; selective outcome reporting; and other bias. Each potential source of bias will be graded as high, low or unclear and a quote from the study report with a justification for our judgement will be provided in the “Risk of bias” table. The risk of bias judgements across different studies for each of the domains listed will be summarized.

### 3.7 Data synthesis

Data from studies judged to be clinically homogeneous will be pooled using Review Manager 5.3 software. Heterogeneity between studies will be assessed using the Cochran's *Q* and Higgins *Ι*^2^ statistic. *P* < .10 for the Chi^2^ statistic or an *I*^2^ > 50% will be considered as showing considerable heterogeneity, and the data will be analyzed using the random-effect model. Otherwise, the fixed-effect model will be used. The Mantel–Haenszel method will be applied for pooling of dichotomous data and results will be presented as relative risk (RR) with their 95% confidence intervals (CIs). Inverse variance method will be used for pooling of continuous data and results will be presented as standardized mean difference (SMD) with their 95% CI.

#### 3.7.1 Subgroup analysis

If data are sufficient, we will conduct subgroup analyses on different histological types: squamous carcinoma and adenocarcinoma. Subgroup analyses will also be performed to explore potential sources of heterogeneity.

#### 3.7.2 Sensitivity analysis

A sensitivity analysis will be performed to confirm whether the pooled results are robust and credible by excluding highly biased studies.

#### 3.7.3 Dealing with missing data

In the condition of missing or unclear data, study authors will be contacted at the eligibility assessment and/or data extraction stage. Secondary publications may be considered as missing data if they have the same study population.

### 3.8 Publication bias

Egger's regression test will be performed to assess the publication bias of the included studies.^[27]^ If there is a publication bias, trim and fill analysis will be performed.

### 3.9 Evidence evaluation

The evidence grade will be determined by using the guidelines of the Grading of Recommendations, Assessment, Development, and Evaluation (GRADE) system, and using 4 levels—high quality, moderate quality, low quality, and very low quality.^[28]^

## 4 Discussion

S-1 is a promising chemotherapy product with good efficacy and acceptable tolerability in various solid tumors, such as advanced gastric cancer,^[29]^ colorectal cancer,^[15]^ esophageal cancer,^[18]^ non-small-cell lung cancer,^[17]^ pancreatic cancer,^[16]^ and head and neck cancer.^[30]^ And as mentioned above, S-1 has also exhibited excellent effects as a CRT regimen for esophageal cancer. This protocol presents the methodology of a systematic review for assessing the efficacy and safety of S-1 combined with radiotherapy in the treatment of advanced unresectable esophageal cancer. We will comprehensively search, screen, assess, and extract valuable data from several databases as previously mentioned, and report this review results according to the PRISMA guidelines. To our knowledge, this will be the first systematic review and meta-analysis comparing S-1-based CRT with radiotherapy alone in the treatment of advanced esophageal cancer regardless patient race.

## 5 Author contributions

**Conceptualization:** F. Liu, Y. Song.

**Data curation:** W. Wang, D. Xing.

**Formal analysis:** W. Wang, D. Xing.

**Funding acquisition:** F. Liu.

**Investigation:** F. Liu.

**Methodology:** W. Wang, D. Xing.

**Project administration:** F. Liu.

**Resources:** D. Xing.

**Software:** W. Wang.

**Supervision:** F. Liu, Y. Song.

**Validation:** Y. Song.

**Visualization:** Y. Song.

**Writing – original draft:** W. Wang, D. Xing.

**Writing – review & editing:** W. Wang, D. Xing.
